# Protective effect of lycopene on oral mucositis and antioxidant capacity of blood plasma in the rat exposed to gamma radiation

**DOI:** 10.22088/cjim.11.4.419

**Published:** 2020

**Authors:** Mina Motallebnejad, Shaghayegh Zahedpasha, Ali Akbar Moghadamnia, Sohrab Kazemi, Daryoush Moslemi, Mahdi Pouramir, Fariba Asgharpour

**Affiliations:** 1 Oral Health Research Center, Health Research Institute, Babol University of Medical Sciences, Babol, Iran; 2 Dental Research Center, Mazandaran University of Medical Sciences, Sari, Iran; 3 Neuroscience Research Center, Health Research Institute, Babol University of Medical Sciences, Babol, Iran; 4 Cellular and Molecular Biology Research Center, Health Research Institute, Babol University of Medical Sciences, Babol, Iran; 5 Department of Radiation Oncology, Babol University of Medical Sciences, Babol, Iran; 6 Department of Clinical Biochemistry, Faculty of Medicine, Babol University of Medical Sciences, Babol, Iran.; 7 Dental Materials Research Center, Health Research Institute, Babol University of Medical Sciences, Babol, Iran

**Keywords:** Antioxidant activity, Irradiation, Lycopene, Mucositis, Radiotherapy

## Abstract

**Background:**

Nowadays, radiotherapy is used effectively for the treatment of head and neck cancers. Mucositis is one of the most important side effects of radiotherapy. Radio-protective agents protect tissues and cells against the adverse effects due to ionizing radiation and cleave radiation-induced free radicals. Lycopene as a potent antioxidant protects cells against oxidative damage by free radical–scavenging. The present study investigated the antioxidant effect of lycopene on oral mucosa of irradiated rats.

**Methods:**

In this experimental animal study, 28 rats were placed in four groups as follows: treated with 50 mg /kg of lycopene (L50), solvent+irradiation (SR), 25 mg / kg of lycopene+irradiation (LR25), and 50 mg / kg of lycopene+irradiation (LR50). The rats received lycopene intraperitoneally. On the irradiation day (day 0) and tenth day of radiation, blood samples were taken from the animals for FRAP and TBARS tests.

**Results:**

The results showed that the LR50 group did not show mucositis higher than grade 2. There was a significant difference (p<0.05) between SR and the L50 regarding the severity of mucositis. In addition, L50 showed higher antioxidant activity and lower peroxidation than SR.

**Conclusion:**

Lycopene reduced the severity of mucositis. Therefore, it can be used as a potential and promising nutritional substance to prevent radiotherapy complications, especially in the treatment of head and neck cancers. However, further research is necessary to confirm these results.

Despite therapeutic advances in recent years, oral cancer has been one of the leading ten causes of mortality due to delayed diagnosis, and recent studies have shown an increase in oral and maxillofacial malignancies (1). Treatments for head and neck cancers include chemotherapy, radiotherapy, and surgery. Oral mucositis is the most painful complication during and after radiotherapy in the head and neck, leading to the interruption of the course of treatment (2). The oral mucositis causes ulceration, burning, erythema and edema in the oral mucosa. Patients receiving chemoradiotherapy due to head and neck tumors or the patients undergoing high doses of chemotherapy prior to bone marrow transplantation typically experience more severe oral mucositis (3). Factors such as the type of treatment and the degree of susceptibility of patients affect the incidence of oral mucositis (3, 4), but there are no effective treatment protocols to reduce oral mucositis (5). Several steps are involved in the development of oral mucositis. 

First, the DNA molecule is damaged by direct irradiation or free radicals, resulting in increased activity of transcription factors and enzymes. Subsequently, inflammatory cytokines such as TNF-α, IL-6, and IL-1β increased and basal and sub-mucosal cells are damaged leading to inflammation and tissue injury and enter the wounded and infectious phase (6, 7). In severe stages of mucositis, the wound covers the entire epithelium and penetrates the submucosa. At this stage, the person feels severe pain (8). Researchers have shown that the use of natural radioprotectors can help treat oral mucositis. These natural agents are likely to have different mechanisms, such as the elimination of free radicals produced during radiotherapy and the protection of globulin proteins, superoxide dismutase (SOD) and glutathione (GSH) (9).

Carotenoids are a group of lipid-soluble non-enzymatic antioxidants that are found in plants, vegetables, red fruits, watermelon, tomatoes, peaches and red grapes. Carotenoids, as free radical scavengers, protect the body against oxidative stress (10-12). Antioxidants play a major role in the prevention and treatment of human cancer. They destroy oxygen free radicals that cause DNA damage and gene mutations (9). Many oncologists believe antioxidants may reduce the risk of cancer and accelerate recovery during chemotherapy and radiotherapy, and decrease the side effects of these treatments (13). 

The lycopene is an isomer of carotene, which showed the highest antioxidant activity among carotenoids to protect cells against free radicals (12). It can suppress cancer cell proliferation through various mechanisms, including cell cycle arrest, the effect on some signaling pathways, inducing apoptosis, increasing intercellular adhesion, and inhibiting adhesion (14). Lycopene, as an anti-inflammatory agent, reduces proinflammatory cytokines expression such as IL-8 and IL-6 and NF-κB (15). The reports indicated that few research studies have been carried out on the radioprotective effect of lycopene. Franco et al. 2012 showed that lycopene significantly reduced radiation-induced skin toxicity in patients undergoing chemotherapy with anthracyclines and taxanes (16). In the study of Srinivasan et al. 2007, the pretreatment with lycopene to gamma-irradiated lymphocytes prevented the cell from γ-radiation induced damage (17). In another study, lycopene protected the liver and small intestine against radiation-induced oxidative effects in rats (18, 19). Also, lycopene supplementation significantly reduced gastrointestinal side effects in rats treated with radiation (20). Therefore, cells are treated with lycopene before exposing them to oxidative stress, it may be an effective method to reduce the adverse effects of radiation byproducts. (21). Due to the pharmacological effects of lycopene, since few studies have been found of the effect of lycopene on radiation-induced oral mucositis, the purpose of this study was to evaluate the clinical effects of lycopene on the process of mucositis and its antioxidant activity in plasma samples of rats. 

## Methods

**Analytical reagents and standards: **The following reagents and standards: 2,4,6-Tris(2-pyridyl)-s-triazine (TPTZ), 6-hydroxy-2,5,7,8-tetramethylchromane-2-carboxylic acid (Trolox), butylated hydroxytoluene (BHT), acetate buffer, trichloroacetic acid (TCA), thiobarbi- turic acid (TBA), hydrogen chloride and ferric chloride were purchased from Sigma-Aldrich (Saint Louis, MO, USA). 

**Agents: **To meet the study objectives, we used concentrated extract of lycopene with purity of 94% in high performance liquid chromatography (HPLC) dissolved in Tween 80. All experiments were carried out under subdued lighting.

**Animal models: ** The Research Ethics Committee of Babol University of Medical Sciences approved this study (mubabol, HRI.REC.1395.17), and recommended for experimental animal research. Based on previous studies, twenty-eight male Wistar albino rats (2.5–3-month-old) with the mean (± SD) body weight of 165.85 ± 4.06 g were used for the experiment. According to the guidelines for laboratory animal care, rats were maintained in a temperature-controlled environment (22± 28) and fed ad libitum with a standard rat diet during the study. The rats were randomly divided into four groups (n= 7 /group), each as follows: L50 (received 50mg/kg of lycopene), SR (received Tween 80 as lycopene solvent + irradiation), LR25 (received 25 mg/kg of lycopene + irradiation) and LR50 (received 50mg/kg of lycopene + irradiation). The groups received different lycopene concentrations for 10 days via IP injection after irradiation. 

**Irradiation: **After weighing, rats were anaesthetized with 80 mg/kg of ketamine (ketamine hydrochloride, Alfesan, Netherlands) and 12 mg/kg of xylazine (xylazine hydrochloride Bayer, Alfesan, Netherlands) prior to irradiation. Their heads were irradiated with a linear accelerator (Elekta Compact™, China, 6MV). The amount of radiation for each rat was 1400 cGy /min, maximum size field (40×40) with SSD of 80, depth of 1.5 and MU of 843.Following the irradiation, the animals were transferred to the Animal Center at Babol University of Medical Sciences and, the groups received lycopene intraperitoneally during 10 days of the study. The lips and tongues of rats were examined daily to assess mucositis according to Parkin’s clinical scale (22). The day of irradiation (day 0) and after 10 days, the blood samples were collected from the rats. At the end of the research, after weighing the rats, the animals were sacrificed inside a carbon dioxide chamber. 

**FRAP assay: **Total antioxidant powers were estimated with the ferric ion reducing antioxidant power (FRAP). The reagent solution was prepared using 25 ml of 300 mM /l acetate buffer (pH 3.6), 2.5 ml of a 10 mmol/l TPTZ (2, 4, 6-tripyridyl-s triazine; Sigma), 40 mmol/l HCl and 2.5 ml of 20 mmol/1 FeCl3. Briefly, 50μl of sample or standard was mixed in a test tube with 1.5 ml of FRAP reagent, and was then vortexed and incubated at 37°C for 10 min in the dark, the absorbance (OD) was measured at 593 nm by spectrophotometer. Using different concentrations of FeSO4 (0 to 1000 μmol/l), the calibration curve was constructed, and the results was expressed as ferrous ion equivalent (23). 

**TBARS values: **Thiobarbituric acid reactive substances (TBARS), as an estimation of lipid peroxidation, were determined by spectrophotometric method. For preparing the solution of TBA /TCA, 0.375 gr of TBA, 15 gr of TCA with 100 ml of HCL (0.25 mol/l) were mixed and heated. 500 μl of samples were added to tubes containing 2 ml of homogenized fish meat (10 gr in 100 ml of distilled water), then mixed with 4 mL of TBA /TCA solution and 50 μl of BHT (0.01%) heated in a constant-temperature water bath at 100°C for 15 min to develop a pink color. The tubes were centrifuged at 3000 rpm for 15 min at room temperature. The OD of surfactant was read at 532 nm.(24). 

**Statistical analysis: **GraphPad Prism v 6.07 (GraphPad Software Inc., La Jolla, CA, USA) was used for data analysis. Intergroup comparison was performed via t-student tests, and the one-way ANOVA was applied to determine any statistically significant differences in the mean levels of evaluated parameters between different groups. A p≤0.05 was considered significant. 

## Results

Based on the results of this experimental animal study, the mean body weight of all groups rose significantly during the experiment (p<0.001), but there was no significant difference between L50 and other groups (SR, LR50 and LR25) (table1). 

**Table 1:** Mean weight of rats at the baseline and after treatment

**Groups (n = 7)**	**Day 0 ** ^*^	**Day 10**
L50	164.86±3.10	181.86±6.35^a^
SR	168.86±5.50	178.43±5.04^ a^
LR25	164.14±2.38	175.29±1.89^ a^
LR50	165.57±3.17	177.71±3.40^ a^

^*^ Day of irradiation, ^a ^There was a significant difference between days 0 and 10 in each group.

**Figure 1:**
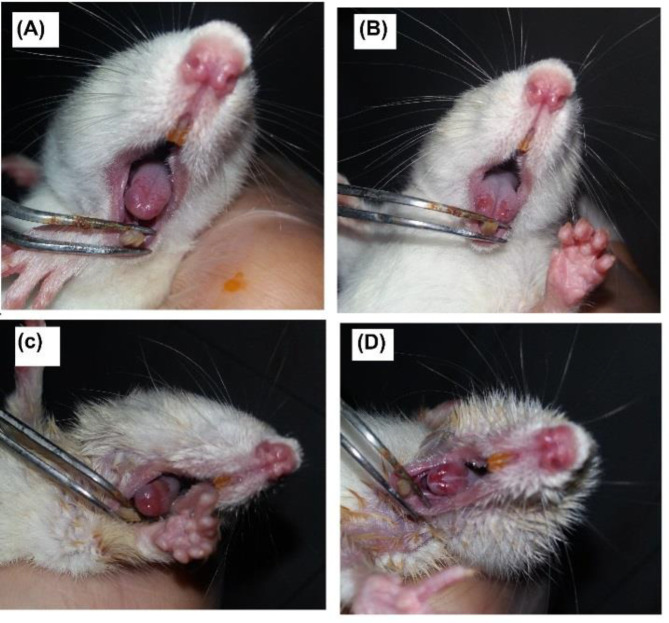
Mucositis according to Parkin’s clinical scale. (A) grad 1, (B) grad 2, (C) grad 3 and (D) grad 5

The rats receiving radiation (SR, LR50 and LR25 groups) were examined daily for tongue and lips with Parkin's clinical scale for mucositis (figure 1). 

After irradiation, one of the LR25 rats died. On day 3 (which was the onset of mucositis in majority of rats), 5 rats in the PR group, all rats in the LR50 group and 4 in the LR25 rats had mucositis with grade 0-2, and 2 rats in the SR group had mucositis with grade 3–5. On day 6 (peak of mucositis), all rats in the SR and LR25 groups had mucositis with grade 3–5, but rats in the LR50 group remained with grade 0–2. On day 10 and by the end of treatment, all rats in the SR and LR25 groups returned to grade 0-2, but only two rats in the LR50 group remained in grade 0-2, and the rest of the rats recovered completely. These results showed that mucositis did not reach more than grade 2 in LR50, and 72.5% of rats were treated by the end of the period (table 2). 

**Table 2:** Distributions of grade 0–2 and grade 3–5 of mucositis examination according to Parkin’s clinical scale

**Groups (n = 7)**	**3days**					**6 days**					**10 days**			
	**Grade 0–2**		**Grade 3– 5**			**Grade 0– 2**		**Grade 3– 5**			**Grade 0–2**		**Grade 3– 5**	
	n	%	n	%		n	%	n	%		n	%	n	%
L50	0	0	0	0		0	0	0	0		0	0	0	0
SR	5	71.4	2	28.5		0	0	7	100		7	100	0	0
LR25	4	57.1	0	0		0	0	6	85.7		6	85.7	0	0
LR50	7	100	0	0		7	100	0	0		2	28.5	0	0

**Figure 2:**
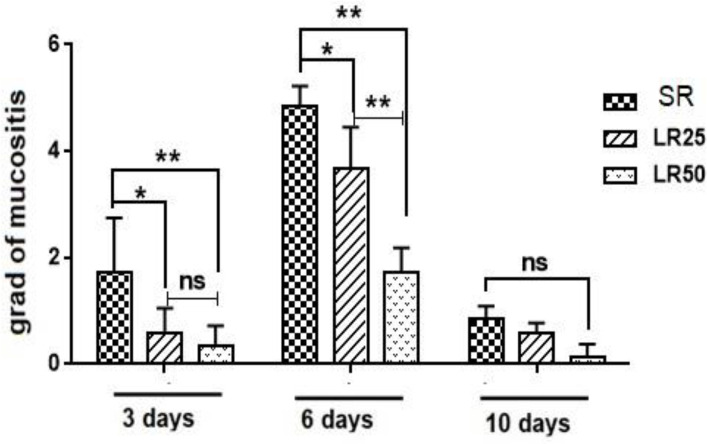
Comparison the mucositis (mean ± SD) in the groups on the 3rd, 6th and 10th days after treatment; * (p <0.05), ** (p <0.001) and ns; non-significant

Figure 2 shows the comparison of the grades between groups on the 3rd, 6th and 10th days. Significant difference was observed in the severity of mucositis on day 3 between the SR and the LR50 and LR25 (p<0.01 and p<0.05, respectively), while no significant difference was observed between the LR25 and LR50 groups. On day 6, there was a significant difference between the LR50 group and the LR25 and SR (p<0.001) (figure 1).

**Figure 3:**
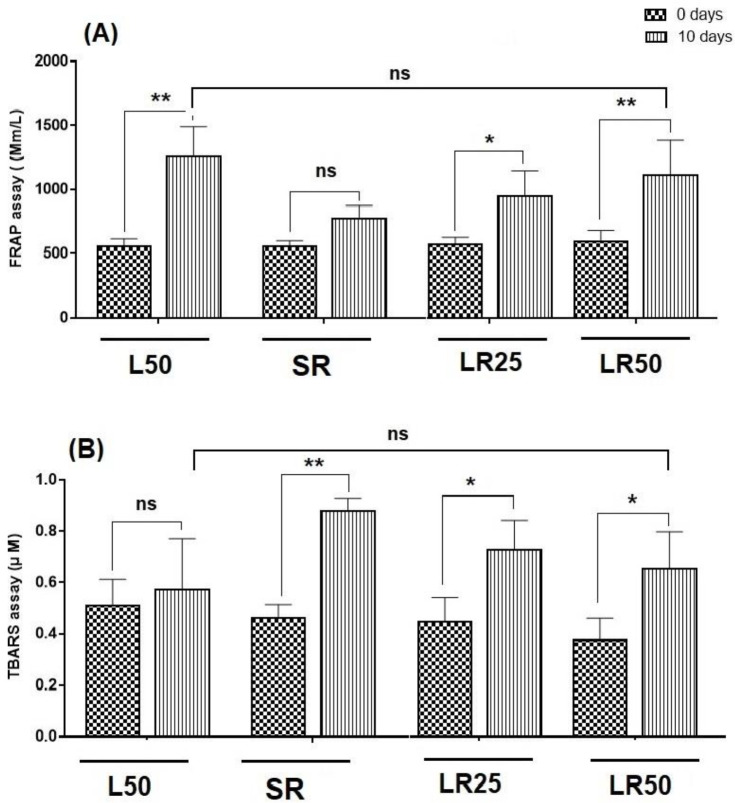
Antioxidant activity of FRAP assay (A) and TBARS assay (B) of rat’s serum on days 0 and 10 of treatment; Result are expressed as mean ± SD; * (p< 0.01), ** (p<0.001) and ns; non-significant

As shown in figure 3A, the FRAP levels in all rats increased after 10 days, but this increase was not significant in the SR group. Comparison of mean 10th day of L50 was significant compared to 10th day of SR and LR25 (p <0.001 and p<0.05, respectively) but this difference was not significant compared to LR50. In the TBARS, there was a significant difference between day 0 and day 10 in the exposed groups. A significant difference was observed in the level of lipid peroxidation in the L50 compared to the SR and LR25 groups (p<0.001 and p<0.05, respectively). These results indicate that the rats in the LR50 group receiving higher concentrations of lycopene showed higher antioxidant production and lower peroxidation levels than SR and LR25 (figure 3B).

## Discussion

In the present study, the rats receiving higher levels of lycopene (LR50) did not show mucositis severity higher than grade 2, and 72.5% were treated by the end of the period. In addition, the rats in the LR50 group showed higher antioxidant production and lower peroxidation levels than the solvent group and the group receiving lower concentration of lycopene (LR25). Lycopene is a potent antioxidant among carotenoids, and as an antioxidant it prevents damage to adipose membrane cells and DNA. Research has shown that administration of lycopene before treatment can protect cells against radioactive damage because it prevents the peroxidation of membrane lipids and free radicals leading to cleavage of DNA strands (25). Lycopene not only is a chemo preventive agent, but also it shows anti-proliferative activity in human and animal cancer cells in lung, breast, and colon cancers. Reports have shown that little research has been done on the protective effect of lycopene against radiation. Coskun et al. 2017 of Turkey investigated the effect of lycopene on the prevention of acute esophageal toxicity caused by radiation in the rat. In this study, histopathological results revealed that lycopene-treated groups had more improvement than the radiation-treated group alone. In addition, the greatest improvement was seen in the group receiving lycopene before and after radiation. Changes in the membrane with grades 2–3 of necrosis and vacuole shape were significantly lower in lycopene-treated groups in comparison with lycopene-free group (26). 

Intraperitoneal administration of lycopene to irradiated rats in the study of Forssberg et. al. 1959, at the University of Stockholm, showed that lycopene administration before X-irradiation protected mice from lethal bacterial infections (27, 28). In other study, the treatment of rats with lycopene (20mg / kg) 24 h before irradiation caused less damage to the structure of the parathyroid glands. They suggested that lycopene could provide protection against the effects of radiation (25). Peak accumulation of radioactivity occurred between 4 and 8 h after gavage dose of [14C] lycopene in rat plasma. (29). The ionizing radiation develops the oxidative stress through the production of ROS, leading to an imbalance of prooxidants and antioxidants in exposed cells (30). Numerous studies have shown that lycopene can improve cellular damage due to reactive oxygen species (ROS)by neutralizing free radicals such as ^1^O2, NO2•, RS• and RSO2• (21,31,32). Srinivasan et al. 2007, indicated that the pretreatment with lycopene in gamma-irradiated lymphocytes resulted in decreased lipid peroxidation and increased activity of GSH, SOD and catalase in the lycopene group compared to the control group. This protective effect of lymphocytes might be attributed to the antioxidant properties of lycopene (17). Also, lycopene indicated a protective effect against lipid peroxidation caused by chemotherapy drugs that had cytotoxic effects similar to irradiation (30, 33). Meydan et al. 2011 of Turkey, investigated oxidative damage of radiation in rat liver tissue. In this study, pretreatment with lycopene attenuated some of the effects of radiation and suggested that dietary supplements such as lycopene decreased radiation-induced liver toxicity by decreasing lipid peroxidation and increasing GSH and SOD activity. (34). In other study, the rats received lycopene (5 mg / kg) by gavage for 7 days before gamma exposure, which resulted in a decrease in the amount of TBARS recorded for each subcellular structure in the irradiated liver (35). In addition, other studies showed that lycopene caused a protective effect on radiation-induced intestinal toxicity via an increase in activity antioxidant enzymes and a decline in lipid peroxidation. (19, 36). The pretreatment with lycopene caused an increase in enzyme activity and a decrease in TBARS levels in the cell organelles compared to cells receiving radiation (RT) alone. Since irradiation causes free radical release and ROS production, and this overproduction of ROS for a few minutes or even days may be responsible for radiation damage in the days following radiation exposure. Therefore, long-term treatment with lycopene can reduce the free radical overproduction (37, 38). It seems that, lycopene is a promising candidate for the protection of radioprotection in normal cells and prevention of cancer development as a natural antioxidant.

In conclusion the current study showed that use of lycopene before and after irradiation reduced the radiation-induced oxidative damage in rats via stimulating antioxidant enzyme activity and reducing lipid peroxidation. The results also indicated that continued treatment with lycopene may be helpful. However, there is a need for further studies to confirm the benefits of lycopene and its application in the prevention of head and neck cancers.
